# Entropy of the Canonical Occupancy (Macro) State in the Quantum Measurement Theory

**DOI:** 10.3390/e26020107

**Published:** 2024-01-24

**Authors:** Arnaldo Spalvieri

**Affiliations:** Dipartimento di Elettronica, Informazione e Bioingegneria, Politecnico di Milano, 20133 Milan, Italy; arnaldo.spalvieri@polimi.it

**Keywords:** occupancy numbers, multivariate hypergeometric distribution, multinomial distribution, canonical typicality, Gibbs correction factor, Sackur-Tetrode entropy formula

## Abstract

The paper analyzes the probability distribution of the occupancy numbers and the entropy of a system at the equilibrium composed by an arbitrary number of non-interacting bosons. The probability distribution is obtained through two approaches: one involves tracing out the environment from a bosonic eigenstate of the combined environment and system of interest (the empirical approach), while the other involves tracing out the environment from the mixed state of the combined environment and system of interest (the Bayesian approach). In the thermodynamic limit, the two coincide and are equal to the multinomial distribution. Furthermore, the paper proposes to identify the physical entropy of the bosonic system with the Shannon entropy of the occupancy numbers, fixing certain contradictions that arise in the classical analysis of thermodynamic entropy. Finally, by leveraging an information-theoretic inequality between the entropy of the multinomial distribution and the entropy of the multivariate hypergeometric distribution, Bayesianism of information theory and empiricism of statistical mechanics are integrated into a common “infomechanical” framework.

## 1. Introduction

The concept of physical entropy is controversial since the times of Boltzmann and Gibbs. The subsequent excerpt from an interview with Shannon, whose crucial contribution to the comprehension of entropy is now widely acknowledged not only in physics [1] but also in diverse fields such as medicine, seismology, and finance (refer to [2,3,4] for recent applications of Shannon entropy in these domains), is available in [5]: “*My greatest concern was what to call it. I thought of calling it ‘information’, but the word was overly used, so I decided to call it ‘uncertainty’. When I discussed it with John von Neumann, he had a better idea. Von Neumann told me, “You should call it entropy, for two reasons. In the first place your uncertainty function has been used in statistical mechanics under that name, so it already has a name. In the second place, and more important, no one really knows what entropy really is, so in a debate you will always have the advantage”.*”

Over the years, the number of different interpretations and definitions of entropy has grown, a recent collection of heterogeneous “entropies” being reported in [6].

In this fragmented and ambiguous context, one of the most debated points is the relationship between information and physical entropy. The controversy about the role of information in physics has its roots in the famous thought experiments of Maxwell and Szilard, and is still an object of discussion today. From the one side, Landauer claims in [7] that

*Information* is a physical entity.

From the other side, Maroney writes in his thesis [8] that

*The Szilard Engine is unsuccessful as a paradigm of the information-entropy link,*

and Maroney and Timpson reiterate the same concept in [9]:

*rejecting the claims that information is physical provides a better basis for understanding the fertile relationship between information theory and physics.*

Independently of the different opinions and arguments, it is a matter of fact that entropy is the key quantity that allows us to derive all the thermodynamic macro-properties of many-particle systems from the statistical properties of the constituent microscopic particles.

One standard approach to entropy in the classical (non-quantum) regime is based on the Gibbs entropy of microstates, which, for systems at equilibrium with the environment, is the Shannon entropy of the probability distribution resulting from entropy maximization under the constraints imposed on the system by the environment that surrounds it. The principle of maximization of entropy of microstates, often referred to as MaxEnt, dates back to the seminal paper of Jaynes [10] and inspired Shannon’s information-theoretic view of entropy. Regrettably, the probability distribution of microstates does not account for indistinguishability of particles. At the same time, indistinguishability of particles is essential; therefore, it is imperative to incorporate it into the framework. This is typically accomplished by subtracting from the entropy of microstates the Gibbsian correction term log(N!), where *N* is the number of the system’s particles.

The Gibbsian correction term enters the scene almost like a sudden intervention, akin to a *deus ex machina*, but the subtraction of log(N!) generates a glaring contradiction: when the entropy of microstates is smaller than log(N!), the difference between the entropy of microstates and log(N!) becomes negative, as it actually happens to the Sackur–Tetrode entropy formula at a low temperature-to-density ratio. Since the entropy of any random variable is guaranteed to always be non-negative (this statement is, according to [11], a formulation of the third law of thermodynamics), entropy of microstates minus log(N!) cannot be entropy. This pathology is not surprising since, after the subtraction of log(N!), the random variable for which the “entropy” is calculated is no longer specified. Consequently, “entropy” transforms into a mere formula representing an unspecified physical system. In this controversial situation, more than 100 years after its introduction, the Gibbsian log(N!) is still an object of research and debate today; see, e.g., [11,12,13,14,15], see also [16] for the case of small thermodynamic systems.

This paper recovers coherency of the picture by considering, in place of the classical setting, the more general quantum setting, where entropy is the von Neumann entropy of the mixed state of the system. The mixed state of a system is commonly represented by a density operator living in the Hilbert space $H^{⊗N}$ obtained as the tensor product *N* times of the single-particle Hilbert space H. Exactly as it happens with the entropy of microstates, the von Neumann entropy of this density operator is appropriate for systems of distinguishable particles, but it does not capture the indistinguishability of particles. We observe that, to capture the indistinguishability of particles of a bosonic system, i.e., a system whose state remains unchanged when particles’ indexes are permuted, an eigenbasis that conveniently represents its quantum state is that of the vectors of the occupancy numbers, giving rise to the so-called second quantization formalism. The consideration of indistinguishability of particles and, with it, of the occupancy numbers, leads us to define the bosonic density operator, i.e., the density operator in a system’s bosonic subspace. Due to the inherent capability of the bosonic density operator to capture the indistinguishability of particles, its von Neuman entropy is the entropy of the system. This allows us to surpass the standard approach to entropy based on distinguishable particles and resolves the puzzle of the Gibbsian log(N!), which, as shown in this paper, now enters the scene through the main entrance alongside other quantum correction terms ensuring non-negativity of entropy.

Our exploration of the quantum approach is situated within the framework of contemporary quantum thermodynamics, whose origins can be attributed to [17,18]. The two cited papers, which have deeply influenced the successive literature, e.g., [19,20,21,22,23], derive the mixed state of a system at equilibrium with the environment by tracing out the environment from the universe, which, in the thermal case, is the union of the system and the heat bath that thermalizes the system. The analysis of [17,18] shows that, as the number of particles of the universe tends to infinity, the mixed state of the system is the same, at least in the weak sense, both if the universe is in a pure state or in a mixed state. Purity of the state of the universe makes the introduction of a statistical ensemble of “universes” unnecessary, thus overcoming the subjectivism that is inherent in the Bayesian approach.

The outline of the paper is as follows. Section 2 introduces the bosonic Hilbert subspace and its eigenbasis. In Section 3, we derive the bosonic density operator for the system under consideration by tracing out the environment from the universe, assuming that the universe is in a bosonic eigenstate. With this assumption, we show that the probability distribution that weights the projectors of the bosonic Hilbert subspace of the system is the multivariate hypergeometric distribution. As the number of bosons of the universe tends to infinity, the multivariate hypergeometric distribution converges to the multinomial distribution, which we identify as the canonical distribution of the occupancy numbers. Section 4 shows that, if, as in the Bayesian approach, the universe is assumed to be in a mixed bosonic state, then the distribution of the occupancy numbers of the system is multinomial provided that the occupancy numbers of the universe are multinomially distributed too. Section 5 discusses the application of the mentioned probability distributions to the entropy of physical systems and places our work within the framework of quantum information theory, unveiling an engaging connection between Bayesianism and empiricism in physics. To illustrate the intrinsic capacity of the canonical bosonic density operator to capture the indistinguishability of particles in systems at equilibrium, Section 6 shows that its von Neumann entropy fits the entropy of the ideal gas in a container. Section 7 sketches the future application of our approach to the Szilard engine. Finally, in Section 8 we draw the conclusions.

## 2. The Bosonic Hilbert Subspace

Let {c∈C}, C={1,2,⋯,|C|}, be the set of quantum numbers (the colors) allowed to a boson, let $\left\{\bar{c}=\left(c_{1},c_{2},\cdots ,c_{N}\right)\right\}$ (the overline denotes vectors and their size is not explicitly mentioned for brevity when it can be inferred from the context) be the set of microstates of a system made by *N* non-interacting bosons (the balls), and let $\left\{|\bar{c}\rangle \right\}$ be the complete set of eigenstates that span the Hilbert space $H^{⊗N}$ of the system. Let δ(·) be the indicator function and let

$$n_{c}\left(c_{1},c_{2},\cdots ,c_{N}\right)\overset{\mathrm{def}}{=}\sum_{i=1}^{N}\delta \left(c-c_{i}\right),\;c=1,2,\cdots ,\left|C\right|,$$

be the number of bosons whose quantum number is *c*. In the following, the dependency on $\left(c_{1},c_{2},\cdots c_{N}\right)$ of $n_{c}\left(c_{1},c_{2},\cdots c_{N}\right)$ will be omitted when possible and the vector of the occupancy numbers $\bar{n}=\left(n_{1},n_{2},\cdots ,n_{\left|C\right|}\right)$ will be called occupancy macrostate. The quantum state of the system of indistinguishable bosons is

(1) $$\begin{array}{c} |\bar{n}\rangle =\sum_{\bar{c}\in C^{N}\left(\bar{n}\right)}\frac{|\bar{c}\rangle }{\sqrt{W(\bar{n})}}; \end{array}$$

see, e.g., [24] and chapter 7 of [25]. In the above equation, $C^{N}\left(\bar{n}\right)$ is the set of the $W(\bar{n})$ equiprobable microstates whose occupancy macrostate is $\bar{n}$ and $W(\bar{n})$ is the multinomial coefficient,

$$\begin{array}{c} W\left(\bar{n}\right)=\left\{\begin{array}{cc} \frac{N!}{\prod_{c=1}^{\left|C\right|}n_{c}!}, & n_{c}\geq 0,\;\forall \;c\in C,\\ 0, & \mathrm{elsewhere}, \end{array}\right. \end{array}$$

i.e., the number of distinct permutations of the entries of any one of the elements of $C^{N}\left(\bar{n}\right)$.

The set $\left\{|\bar{n}\rangle ,\bar{n}\in N\right\}$, with

$$\begin{array}{c} \left|N\right|=\left(\begin{array}{c} N+|C|-1\\ |C|-1 \end{array}\right) \end{array}$$

(see [26]) is a complete set of bosonic eigenstates for the bosonic Hilbert subspace of the Hilbert space of the system.

## 3. Empirical Approach

We want to obtain the system’s bosonic density operator ${\hat{\nu }}_{\bar{u}}$, where $\bar{u}$ is the occupancy macrostate of the universe, tracing out the environment made by U−N bosons from the projector $|\bar{u}\rangle \langle \bar{u}|$ of the universe made by *U* bosons. The assumption that the universe is in an eigenstate appears to be plausible, especially for large values of *U* and within the context of the Eigenstate Thermalization Hypothesis (ETH) [27]. The first step is to write $|\bar{u}\rangle$ in the form of bosonic purification:

$$\begin{array}{cc} |\bar{u}\rangle & =\sum_{\bar{n}\in N,}\sum_{{\bar{c}}_{1}^{N}\in C^{N}\left(\bar{n}\right),}\sum_{{\bar{c}}_{N+1}^{U}\in C^{U-N}\left(\bar{u}-\bar{n}\right)}\frac{|{\bar{c}}_{1}^{N},{\bar{c}}_{N+1}^{U}\rangle }{\sqrt{W(\bar{u})}}\\ \; & =\sum_{\bar{n}\in N}\sqrt{\frac{W\left(\bar{n}\right)W\left(\bar{u}-\bar{n}\right)}{W(\bar{u})}}|\bar{n},\bar{u}-\bar{n}\rangle , \end{array}$$

where ${\bar{c}}_{i}^{j}$ represents the windowing of vector $\bar{c}$ between *i* and *j* and it is understood that the set $C^{N}\left(\bar{n}\right)$ ($C^{U-N}\left(\bar{u}-\bar{n}\right)$) is empty when one or more entries of $\bar{n}$ ($\bar{u}-\bar{n}$) is negative. Observing that

$$\langle \bar{u}-\bar{n}|\bar{u}-{\bar{n}}^{\prime }\rangle |\bar{n}\rangle \langle {\bar{n}}^{\prime }|=\left\{\begin{array}{cc} \;|\bar{n}\rangle \langle {\bar{n}}^{\prime }|, & \;\bar{u}-\bar{n}=\bar{u}-{\bar{n}}^{\prime },\\ \;0, & \;\bar{u}-\bar{n}\neq \bar{u}-{\bar{n}}^{\prime }, \end{array}\right.$$

and that the condition $\bar{u}-\bar{n}=\bar{u}-{\bar{n}}^{\prime }$ forces $\bar{n}={\bar{n}}^{\prime }$, we conclude that

(2) $$\begin{array}{cc} {\hat{\nu }}_{\bar{u}} & ={\mathrm{Tr}}_{\;H^{⊗(U-N)}}|\bar{u}\rangle \langle \bar{u}|\\ \; & =\sum_{\bar{n}\in N}\frac{W\left(\bar{u}-\bar{n}\right)W\left(\bar{n}\right)}{W(\bar{u})}|\bar{n}\rangle \langle \bar{n}| \end{array}$$

where ${\mathrm{Tr}}_{\;H^{⊗(U-N)}}$ is the operator that traces out the Hilbert space of the environment. If the set $C^{N}\left(\bar{n}\right)$ ($C^{U-N}\left(\bar{u}-\bar{n}\right)$) is empty, then one or more entries of $\bar{n}$ ($\bar{u}-\bar{n}$) is negative; in which case, the multinomial coefficient $W(\bar{n})$ ($W(\bar{u}-\bar{n})$) is zero by definition. The fraction appearing in (2) is the multivariate hypergeometric distribution, which is the distribution of the occupancy numbers of colors in drawing without replacement *N* balls out of an urn containing *U* balls with color occupancy numbers $\bar{u}$. In many textbooks and papers, the multivariate hypergeometric distribution is expressed by binomial coefficients, as in the equation

(3) $$\begin{array}{c} P_{\bar{u}}\left(\bar{n}\right)=\frac{W\left(\bar{u}-\bar{n}\right)W\left(\bar{n}\right)}{W(\bar{u})}=\frac{\prod_{c=1}^{\left|C\right|}\left(\begin{array}{c} u_{c}\\ n_{c} \end{array}\right)}{\left(\begin{array}{c} U\\ N \end{array}\right)}, \end{array}$$

where P(·) is the probability of the random variable inside the round brackets and ${\;}_{\bar{u}}$ in the subscript means that the probability distribution $\left\{P_{\bar{u}}\left(\bar{n}\right)\right\}$ depends on the vector of known parameters $\bar{u}$.

In the thermodynamic limit, the multivariate hypergeometric distribution converges to the multinomial distribution, i.e., the distribution of the occupancy numbers of colors in drawing with replacement *N* times a ball out of an urn containing colored balls with relative frequency of color *c* equal to P(c):

 limU→∞Pu¯(n¯)=W(n¯)limU→∞W(u¯−n¯)W(u¯)(4)=W(n¯)limU→∞∏c=1|C|(U−1uc)nc(5)=W(n¯)∏c=1|C|(P(c))nc,

$$\begin{array}{c} \lim_{U\to \infty }{\hat{\nu }}_{\bar{u}}=\hat{\nu }=\sum_{\bar{n}\in N}W\left(\bar{n}\right)\prod_{c=1}^{\left|C\right|}{\left(P\left(c\right)\right)}^{n_{c}}|\bar{n}\rangle \langle \bar{n}|, \end{array}$$

where (4) is Stirling’s formula, while, by regarding $\bar{u}$ as the result of a PVM operated on the universe, Equation (5) is recognized to be the Law of Large Numbers (LLN) for the empirical one-particle distribution. Concentration inequalities that bound the probability of deviations of the empirical probability from its expectation, that is the probability of occurrence of non-typical bosonic eigenstates of the universe, can be found in [28] for hypergeometrically distributed random variables, in [29] for multinomially distributed random vectors. Paper [30] demonstrates that the multinomial distribution is the maximum entropy distribution of the occupancy numbers with constrained one-particle distribution. See also [31,32] for the multinomial distribution in statistical mechanics.

By equiprobability of the disjoint microstates belonging to the same occupancy macrostate, we see that, in (3),

$$\begin{array}{c} \frac{W(\bar{u}-\bar{n}\left(c_{1},c_{2},\cdots ,c_{N}\right))}{W(\bar{u})}=P_{\bar{u}}\left(c_{1},c_{2},\cdots ,c_{N}\right). \end{array}$$

Clearly

$$\begin{array}{cc} \lim_{U\to \infty }P_{\bar{u}}\left(c_{1},c_{2},\cdots ,c_{N}\right) & =\prod_{c=1}^{\left|C\right|}{\left(P\left(c\right)\right)}^{n_{c}\left(c_{1},c_{2},\cdots ,c_{N}\right)}\\ & =\prod_{i=1}^{N}P\left(c_{i}\right), \end{array}$$

which explicitly shows that, in the thermodynamic limit, microstates outcoming from the mentioned PVM become independent and identically distributed random variables. What happens is that the LLN cancels the dependencies inside the joint distribution of microstates induced by the constraints imposed on the colors of the *N* balls by the occupancy numbers $\bar{u}$ of the universe in drawing without replacement. Furthermore, the probability distribution $\left\{\prod_{i=1}^{N}P\left(c_{i}\right)\right\}$ also becomes independent of the specific result $\bar{u}$ of the PVM operated on the universe, in the sense that the empirical one-particle distribution $\left\{U^{-1}u_{c}\right\}$ for U→∞ is the same for almost every bosonic eigenstate of the universe, i.e., for the typical bosonic eigenstates of the universe. The one-particle distribution and, with it, the product $\left\{\prod_{i=1}^{N}P\left(c_{i}\right)\right\}$, will depend on the physical constraints imposed on the universe. When the constraint is the temperature, it is widely recognized that $\left\{\prod_{i=1}^{N}P\left(c_{i}\right)\right\}$ is the Boltzmann distribution, which can be found by Jaynes’ constrained maximization of entropy [10].

**Figure 1:**
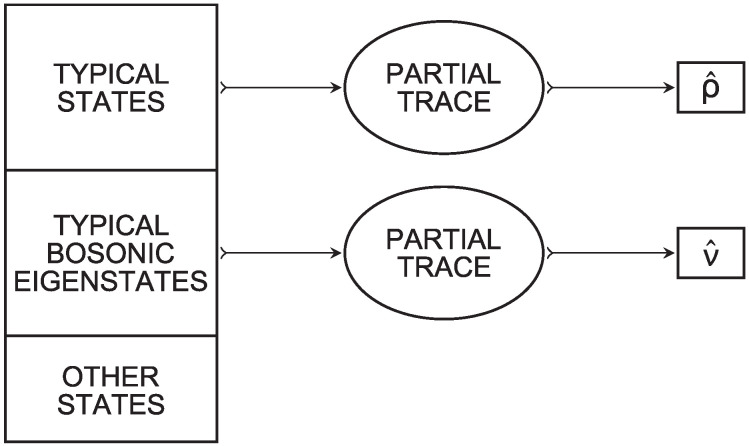
The box on the left represents the pure states of the universe. Although in the thermodynamic limit almost every pure state of the universe is typical, still there is space for infinitely many typical bosonic eigenstates and for infinitely many other pure states that are neither typical states nor typical bosonic eigenstates, among which are all the eigenstates of $H^{⊗U}$.

Independency and identical distribution of the eigenstates of the individual bosons of the system lead to the following density operator in $H^{⊗N}$

$$\begin{array}{cc} \hat{\rho } & =\sum_{\bar{c}\in C^{N}}\prod_{i=1}^{N}P\left(c_{i}\right)|\bar{c}\rangle \langle \bar{c}|, \end{array}$$

which we claim to be the canonical density operator in $H^{⊗N}$. Papers [17,18] claim that the system’s density operator in $H^{⊗N}$ converges to the canonical density operator as U→∞ for almost every pure state of the universe, i.e., for the typical pure states of the universe. For this reason convergence to the canonical state is called *canonical typicality* in [18]. Typicality, which can be intended in various senses, is often invoked in statistical mechanics. For instance, paper [33] uses the properties of the information-theoretic typical set to characterize weak convergence of microstates to equiprobability in the context of classical statistical mechanics. We point out that the claim of [17,18] is compatible with our claim of convergence to the bosonic canonical state if we do not pretend that typical bosonic eigenstates are typical states, as illustrated in Figure 1.

## 4. Bayesian Approach

In the Bayesian approach, the vector of known parameters $\bar{u}$ becomes a random vector whose probability distribution, the Bayesian prior, characterizes the bosonic density operator of the universe. In the Bayesian setting, the hypergeometric multivariate distribution of the density operator ${\hat{\nu }}_{\bar{u}}$ of (2) is the Bayesian likelihood, i.e., a conditional distribution where the random condition is the random vector of parameters $\bar{u}$. The probability distribution of the density operator of the system, the Bayesian marginal, is obtained by tracing out the environment from the bosonic density operator of the universe. The Bayesian approach is self-consistent when the prior is multinomial because the marginal turns out to be multinomial too, regardless of the number of particles of the universe:

 ν^=∑u¯∈UW(u¯)∏c=1|C|(P(c))ucTrH⊗(U−N)|u¯〉〈u¯|  1=∑u¯∈UW(u¯)∏c=1|C|(P(c))ucν^u¯(6)=∑n¯∈NW(n¯)∏c=1|C|(P(c))nc|n¯〉〈n¯|,

where the third equality is obtained by substituting (2) in the second line and manipulating. The comparison between the Bayesian (6) and the empirical (2) clarifies the relationship between the two approaches. The Bayesian approach captures the pre-measurement randomness of the mixed state of the universe by a mathematical procedure based on constrained entropy maximization whose result is the multinomial prior of the universe. Instead, the empirical approach renounces to mathematics and imports the result $\bar{u}$ of the measurement of the occupancy numbers of the universe in the multivariate hypergeometric distribution of the occupancy numbers of a system that is separated from the universe after the measurement.

## 5. Quantum Entropy and Quantum Information

Since ${\hat{\nu }}_{\bar{u}}$ ($\hat{\nu }$) is an eigendecomposition, its von Neumann entropy is equal to the Shannon entropy of the multivariate hypergeometric (multinomial) distribution. Specifically, when the density operator is an eigendecomposition, the associated density matrix is diagonal, leading to

$$S\left(\hat{\nu }\right)\overset{\mathrm{def}}{=}-\mathrm{Tr}\left(\hat{\nu }\log\left(\hat{\nu }\right)\right)=-\sum_{\bar{n}\in N}P\left(\bar{n}\right)\log\left(P\left(\bar{n}\right)\right),$$

where S(·) is the von Neumann entropy of the density operator inside the round brackets and the rightmost term is the Shannon entropy. For this reason, in the following, we make no distinction between the von Neumann entropy and the Shannon entropy, calling it simply “entropy”. It is worth emphasizing that, here, we completely skip the notion of phase space, leading to the exact probability distribution of the quantum occupancy numbers, and, as a consequence, to the exact entropy. Conversely, the standard phase space approach inherently leads to approximations of entropy. These approximations require improvement at low temperature/density ratios, see e.g., [34], while still retaining some approximations. See also [11] for the role of the multinomial coefficient in the solution of the Gibbs paradox in the phase-space approach.

The entropy of the entanglement between the environment and the system of interest is the entropy of the multivariate hypergeometric distribution, which, owing to the distribution’s symmetry, is equal to the entropy of both the system and the environment after their separation. It is

 S(ν^u)=log(W(u¯))−E{log(W(u¯−n¯)}−E{log(W(n¯))}(7)=−E{log(Pu¯(c¯))}−log(N!)+∑c=1|C|E{log(nc!)},

where E{·} is the classical expectation computed over the probability distribution of the random variable inside the expectation, the base of the logarithm is Euler’s number, and the Boltzmann constant in front of the logarithm is omitted for brevity. The term $-E\{\log\left(P_{\bar{u}}\left(\bar{c}\right)\right)\}$ is the Gibbs entropy of microstates, i.e., the entropy of the system of distinguishable particles, while the term $-E\{\log\left(W\left(\bar{n}\right)\right)\}$ is due to indistinguishability of particles, which, in the average case, prevents access to $E\{\log\left(W\left(\bar{n}\right)\right)\}$ units of information. Since ${\left(W\left(\bar{n}\right)\right)}^{-1}$ is the conditional probability of microstates given the macrostate, its expectation is the conditional Shannon entropy of microstates given the macrostate; so, $S({\hat{\nu }}_{u})$ is the mutual information between microstates and macrostates. The term log(N!) in (7) was introduced by Gibbs to force compatibility between the non-quantized phase-space (differential) entropy of microstates and the physical entropy of systems of indistinguishable particles. We observe that, while the probability that two or more classical particles have the same position and momentum in the phase space is zero because position and momentum are dense variables, the probability that two or more quantum particles occupy the same quantum state is not zero. This non-zero probability is captured by the sum of expectations in (7). As the entropy of microstates becomes lower and lower, this sum moves closer and closer to log(N!), until becoming equal to log(N!) when all the particles occupy the ground state. This also prevents the system’s entropy from becoming negative when the entropy of microstates becomes vanishingly small.

In the canonical case, the entropy of the bosonic density operator is the entropy of the multinomial distribution:

(8) $$\begin{array}{cc} \; & S\left(\hat{\nu }\right)=-NE\left\{\log\left(P\left(c\right)\right)\right\}-E\left\{\log\left(W\left(\bar{n}\right)\right)\right\}. \end{array}$$

See [35,36] for the calculation of the above entropy and see [32] for approximations of the entropy of the multinomial distribution in the context of statistical mechanics. Equation (8) is Equation (11) of [32], where the authors call the entropy of the distribution of the occupancy numbers *entropy fluctuations*. Apart from certain exceptions, the authors of [32] consider these entropy fluctuations negligible compared to the entropy of the system, failing to recognize that the entropy of the occupancy numbers is the entropy of a system of indistinguishable particles.

Let us consider the thermal state. The one-particle Boltzmann distribution for a system at the thermal equilibrium at temperature *T* Kelvin degrees is

$$P\left(c\right)=Z^{-1}e^{-\epsilon_{c}/k_{B}T},$$

where $\epsilon_{c}$ is the energy eigenvalue of eigenstate |c〉, $k_{B}=1.38\cdot 10^{-23}$ J/K is the Boltzmann constant and *Z* is the one-particle partition function

$$Z=\sum_{c=1}^{\left|C\right|}e^{-\epsilon_{c}/k_{B}T},$$

where |C| is large enough to make the impact of the truncation of the sum negligible. In the non-quantum regime, where the approximation

$$E\left\{\log\left(W\left(\bar{n}\right)\right)\right\}\approx \log\left(N!\right)$$

holds, the entropy $S(\hat{\nu })$ of (8) fits the classical relationship between thermodynamic entropy and temperature:

 ∂S(ν^)N∂E{ϵc}≈−∂∂E{ϵc}E{log(P(c))} =∂∂E{ϵc}E{log(Zeϵc/kBT)}(9)=1kBT,

where it easy to see that

$$\begin{array}{c} \frac{\partial \log(Z)}{\partial E\{\epsilon_{c}\}}=-E\left\{\epsilon_{c}\right\}\frac{\partial }{\partial E\{\epsilon_{c}\}}\frac{1}{k_{B}T}. \end{array}$$

In the impossibility, for the reasons already discussed, to take the quantum entropy of microstates $S(\hat{\rho })$ and/or $S\left(\hat{\rho }\right)-\log\left(N!\right)$ as candidates to the role of quantum thermodynamic entropy, the right hand side of (8), despite the deviation of its partial derivative w.r.t. the expected energy from (9) in the quantum regime, is a solid candidate for the quantum thermodynamic entropy. Actually, the equality between the partial derivative of the classical thermodynamic entropy w.r.t. the expected energy and (9) is a genuinely classical equality that, due to the quantum effects that become important at a low temperature, can be no more true when the quantum thermodynamic entropy is considered. Also, the expectation of the logarithm of the multinomial coefficient can make (8) non-extensive when the probability that two or more particles occupy the same quantum state is non-negligible; see [37] for non-extensivity of (8) in the case of a monoatomic gas at thermal equilibrium in a container. Again, extensivity of thermodynamic entropy is a genuinely classical concept, so we should not be surprised that there is a lack of extensivity when quantum effects are non-negligible.

A digression is now necessary in order to delve into the relationship between our entropy of macrostates expressed by Equations (7) and (8), and the observational entropy investigated in [6] and in other papers of the same author published from 2019 to the present day, including the recent [38]. Even if the authors of [6] attribute to their observational entropy a meaning that is clearly different from the meaning of our entropy of macrostates, to a certain extent, the two risk being confused, at least formally, because the observational entropy includes a term that is exactly the same as that for our entropy of macrostates. Precisely to avert this risk, we hereafter show that, in our setting, the observational entropy is equal to the Gibbs entropy of microstates. Therefore, its meaning cannot be that of the entropy of macrostates expressed by Equations (7) and (8). Hereafter, the vector $\left(i_{1},i_{2},\cdots ,i_{n}\right)$ appearing in the definition of observational entropy of multiple coarse graining given in Equation (19) of [6] is identified with our $\bar{n}=\left(n_{1},n_{2},\cdots ,n_{\left|C\right|}\right)$. Thus, the observational entropy defined in Equation (19) of [6] is

(10) $$-\sum_{\bar{n}}p_{\bar{n}}\log\left(\frac{p_{\bar{n}}}{V_{\bar{n}}}\right).$$

It is formally admissible and it seems aligned with the spirit of [6] to take for the projector ${\hat{P}}_{n_{1}}{\hat{P}}_{n_{2}}\cdots {\hat{P}}_{n_{\left|C\right|}}$ of [6]

$${\hat{P}}_{n_{1}}{\hat{P}}_{n_{2}}\cdots {\hat{P}}_{n_{\left|C\right|}}={\hat{P}}_{\bar{n}}=\sum_{\bar{c}\in C^{N}\left(\bar{n}\right)}|\bar{c}\rangle \langle \bar{c}|,$$

leading to

$${\hat{P}}_{\bar{n}}{\hat{P}}_{\bar{n}}={\hat{P}}_{\bar{n}}$$

and, by the definition of $V_{\bar{n}}$ given in [6], to

$$V_{\bar{n}}=\mathrm{Tr}\left({\hat{P}}_{\bar{n}}\right)=W\left(\bar{n}\right),$$

that, in full accordance with the spirit of [6], is the dimension of the subspace of $H^{⊗N}$ occupied by the macrostate $\bar{n}$. Using the definition of the probability $p_{\bar{n}}$ of macrostate $\bar{n}$ given in [6], with

$$\hat{\rho }=\sum_{\bar{c}\in C^{N}}P\left(\bar{c}\right)|\bar{c}\rangle \langle \bar{c}|,$$

we get

$$p_{\bar{n}}=\mathrm{Tr}\left({\hat{P}}_{\bar{n}}\hat{\rho }{\hat{P}}_{\bar{n}}\right)=W\left(\bar{n}\right)P\left(\bar{c}\right),\;\;\forall \;\bar{c}\in C^{N}\left(\bar{n}\right),$$

which is equal to the probability of macrostate $\bar{n}$ proposed in this paper and is actually an alternative way to define it, though. Note the difference; we derived it from the partial trace operated on the eigenstate of the universe and/or from the MaxEnt principle. Plugging $p_{\bar{n}}$ and $V_{\bar{n}}$ in (10), from our Equations (7) and/or (8), we conclude that the observational entropy (10) is equal to the Gibbs entropy of microstates. This is enough to avert the risk of confusion between our entropy of macrostates and the observational entropy, because the only case where the entropy of macrostates is equal to the observational entropy occurs when $V_{\bar{n}}=1$ for all the vectors $\bar{n}$, i.e., when particles are distinguishable and the entropy of macrostates is the Gibbs entropy of microstates.

The following inequalities sandwich the Boltzmann entropy $\log(W\left(E\left\{\bar{n}\right\}\right))$ between the two terms in the right-hand side of (8):

(11) $$\begin{array}{cc} -NE\{\log(P(c))\} & \geq \log(W\left(E\left\{\bar{n}\right\}\right))\\ \; & \geq E\{\log\left(W\left(\bar{n}\right)\right)\}, \end{array}$$

where, with some abuse of notation, the factorials of the real numbers in the denominator of $W(E\left\{\bar{n}\right\})$ are intended as x!=Γ(x+1), where Γ(·) is the Gamma function. The first inequality is (11.22) of [26], the second inequality is obtained by applying the Jensen inequality

$$E\left\{f\left(n_{c}\right)\right\}\geq f\left(E\left\{n_{c}\right\}\right),\;\forall \;c\in C,$$

to the convex (upward) function $f\left(n_{c}\right)=\log\left(n_{c}!\right)$. In statistical mechanics, it is standard to derive from Stirling’s formula an approximation between the two terms of (11).

Note that, if we pretend that entropy is a variable of state, then the probability distribution of the occupancy numbers must depend only on the state of the system. However, the multivariate hypergeometric distribution depends also on the state of the universe. The dependency becomes progressively weaker as the number of particles in the universe approaches infinity. However, it remains evident that this makes the empirical approach incompatible with the notion of entropy as a state variable. In conclusion, entropy can only be considered a variable of state if we embrace the Bayesian approach.

We hereafter introduce the ”empirical” information, sketching a new engaging connection between the Bayesian and the empirical approaches to information in quantum measurement theory. Let us regard the PVM operated on the universe as a POVM operated on the system, with the environment in the role of ancilla. The difference between the entropy of the multinomial Bayesian marginal and the expectation over the multinomial Bayesian prior $\left\{P\left(\bar{u}\right)\right\}$ of the entropy of the multivariate hypergeometric Bayesian likelihood of the system is equal to the Holevo upper bound χ above the accessible quantum information that any POVM can achieve [39]:

$$\begin{array}{cc} & \chi =S\left(\hat{\nu }\right)-\sum_{\bar{u}\in U}P\left(\bar{u}\right)S\left({\hat{\nu }}_{\bar{u}}\right)\geq 0. \end{array}$$

Regardless of the value of *U*, in place of the above Bayesian information, one could consistently consider the following empirical information, which does not need the definition of a prior:

(12) $$S\left({\hat{\nu }}_{\mathrm{emp}}\right)-S\left({\hat{\nu }}_{\bar{u}}\right)\geq 0,$$

where $S({\hat{\nu }}_{\mathrm{emp}})$ is the Shannon entropy of the empirical multinomial distribution $\left\{P_{\mathrm{emp}}\left(\bar{n}\right)\right\}$ of the occupancy numbers of the system,

$$P_{\mathrm{emp}}\left(\bar{n}\right)=W\left(\bar{n}\right)\prod_{c=1}^{\left|C\right|}{\left(U^{-1}u_{c}\right)}^{n_{c}}.$$

In (12), the one-particle probability distribution $\left\{U^{-1}u_{c}\right\}$ is the same for the multivariate hypergeometric distribution and for the empirical multinomial distribution. Therefore, the inequality is guaranteed by the maximum entropy property of the multinomial distribution demonstrated in [30]. The difference (12) is the “empirical information” brought by the PVM operated on the universe about the system. As U→∞, both $S({\hat{\nu }}_{\bar{u}})$ and $S({\hat{\nu }}_{\mathrm{emp}})$ tend to $S(\hat{\nu })$ and both the Bayesian information and the empirical information tend to zero. If, after the POVM, a PVM is operated on the system, the total information brought by the two measurements is $S(\hat{\nu })$ in the Bayesian approach and $S({\hat{\nu }}_{\mathrm{emp}})$ in the empirical approach. As U→∞, the total empirical information becomes equal to the total Bayesian information.

## 6. Entropy of the Ideal Gas in a Container

In the case of an ideal monoatomic gas in a cubic container of side *L*, one particle of the gas is modelled as a quantum ”particle in a box” with three degrees of freedom, whose energy eigenvalues with aperiodic boundary conditions are

$$\epsilon_{c}=\left(c_{x}^{2}+c_{y}^{2}+c_{z}^{2}\right)\frac{h^{2}}{8mL^{2}},$$

where *c* consists of the three quantum numbers $\left(c_{x},c_{y},c_{z}\right)$, *m* is the mass of the particle and $h=6.626\cdot 10^{-34}\;\;J\cdot s$ is the Planck constant.

When the temperature-to-density ratio is high, it becomes possible to employ two approximations. In the first one, the partition function is approximated to an integral, see Equation (19.54) of [40], leading to

$$\begin{array}{cc} -NE\{\log(P(c))\} & \approx \frac{3N}{2}\left(1+\log\left(\frac{2\pi mk_{B}TL^{2}}{h^{2}}\right)\right). \end{array}$$

In the second one, with the idea that the probability that two or more particles occupy the same state is negligible, the denominator of the multinomial coefficient is ignored and, for large number of particles, the logarithm of the numerator log(N!) is approximated to Nlog(N)−N, leading to

$$\begin{array}{cc} -E\{\log\left(W\left(\bar{n}\right)\right)\} & \approx -N\log(N)+N. \end{array}$$

Plugging the two approximations in (8), one gets the textbook Sackur–Tetrode formula:

$$\begin{array}{c} S\left(\hat{\nu }\right)\approx N\left(\log\left(\frac{L^{3}}{N}{\left(\frac{2\pi mk_{B}T}{h^{2}}\right)}^{\frac{3}{2}}\right)+\frac{5}{2}\right). \end{array}$$

Note that, as already mentioned in the introduction, the exact entropy of the multinomial distribution (8) is guaranteed to be non-negative, while the Sackur–Tetrode formula becomes negative at low temperature-to-density ratios, where the two mentioned approximations do not hold; see [37,41] for details.

## 7. Future Work

We hereafter sketch the application of our proposed approach to the state of Szilard engine after the insertion of the piston, leaving the complete analysis of the Szilard cycle to future work. In the case of *N* particles, after the insertion of the piston, the number *b* of particles that are found in one of the two sub-containers, say the one of volume $V^{\prime }$, is a binomial random variable $\left(N,V^{\prime }/V\right)$, where *V* is the total volume. The probability distribution of the occupancy numbers is

$$\begin{array}{c} P\left({\bar{n}}^{\prime },{\bar{n}}^{\prime \prime },b\right)=P\left({\bar{n}}^{\prime },{\bar{n}}^{\prime \prime }|b\right)P\left(b\right)=P\left({\bar{n}}^{\prime }|b\right)P\left({\bar{n}}^{\prime \prime }|b\right)P\left(b\right), \end{array}$$

where $\left\{P\left({\bar{n}}^{\prime }|b\right)\right\}$ ($\left\{P\left({\bar{n}}^{\prime \prime }|b\right)\right\}$) is the probability distribution of the occupancy numbers of a gas with *b* (N−b) particles in the sub-container of volume $V^{\prime }$ ($V-V^{\prime }$). For N=1 and $V^{\prime }=V/2$, the entropy after the insertion of the wall is

$$\begin{array}{c} \log\left(2\right)-\sum_{c=1}^{\left|C\right|}P\left(c\right)\log\left(P\left(c\right)\right), \end{array}$$

where the famous log(2) of Landauer [42] comes from the binary equiprobable random variable *b* and {P(c)} is the probability distribution of one particle in a box of volume $V^{\prime }$. We have evaluated the partition function of the Boltzmann distribution with the parametrization of [43], that is the mass of the particle $m=9.11\cdot 10^{-31}$ kg, temperature T=300 K, and one-dimensional box of size $L=20\cdot 10^{-9}$ m. We obtain that the entropy of the single particle with one degree of freedom before the insertion of the piston is 1.988 in $k_{B}$ units. After the insertion of the piston, the size of the one-dimensional box is equal to $10\cdot 10^{-9}$ m. The entropy of one particle in this box is, in $k_{B}$ units, equal to 1.243. Considering also the log(2) that comes from the binary equiprobable random variable, the entropy fall due to the insertion of the piston results equal to 1.988−0.693−1.243=0.052, in excellent agreement with the entropy fall shown in Figure 3 of [43], where the result is derived by the phase space approach.

## 8. Conclusions

Entropy is a macroscopic property of a physical system and, at the same time, it is a mathematical property of randomness. As such, it must be a property of the randomness of system’s macrostates. However, despite the necessity of introducing the somewhat ad hoc term −log(N!) as a kind of *deus ex machina*, from Boltzmann to the present day, the ”entropy” of a system at equilibrium is commonly intended as the entropy of microstates, be it the Gibbs entropy or, to some extent, the Boltzmann entropy $\log(W\left(E\left\{\bar{n}\right\}\right))$. This author has come to the conclusion that the misunderstanding arises from the following two conceptual errors.

The first error consists in regarding $\log(W\left(\bar{n}\right))$ as the “entropy of a macrostate”. Even an author as exceptionally deep as Jaynes writes the following in [44]: *To emphasize this, note that a “thermodynamic state” denoted by $X=\{X_{1},\cdots ,X_{n}\}$ defines a large class C(X) of microstates compatible with X. Boltzmann, Planck, and Einstein showed that we may interpret the entropy of a macrostate as S(X)=klog(W(C)), where W(C) is the phase volume occupied by all the microstates in the chosen reference class C.* This misconception is pervasive in the entire statistical mechanics. The influential authors of [45] attribute to Einstein the idea that an individual macrostate can have entropy: *In fact, already Einstein (1914, Equation (4a)) argued that the entropy of a macro state should be proportional to the log of the “number of elementary quantum states” compatible with that macro state…*. But entropy is a property of a statistical ensemble; therefore, one individual macrostate cannot have entropy. Moreover, if by entropy of the macrostate we mean the entropy of the microstates that constitute it, then we must acknowledge that this entropy has no physical significance because the *elementary quantum state compatible with that macrostate* is physically meaningless when particles are indistinguishable. This misconception is so widespread that it can be found also in standard textbooks. We hereafter quote a passage from the introduction to chapter 16 of [40]: *specification of a macrostate constitutes incomplete information*. In the absence of a formal definition of information, this statement risks becoming misleading. Actually, we have shown that the entropy of the occupancy numbers is the complete information about the system because the entropy of microstates belonging to the same macrostate, due to indistinguishability of particles, is not informative, as it is subtracted in (8) from the entropy of microstates.

The second error is the lack of consideration of the absolute randomness of macrostates. Navigating through the literature of statistical mechanics, you may come across passages like the one of [31] that is hereafter reported: *A crucial observation in statistical mechanics is that the distribution of all macrostate variables gets sharply peaked and narrow as system size N increases. … In the limit N→∞, the probability of measuring a macrostate becomes a Dirac delta…*. Clearly, the quoted statement is wrong because the absolute width of the probability distribution of the occupancy numbers, and, more generally, of any macroscopic observable, such as the system’s energy, becomes broader and broader as the number of particles grows, while, by the LLN, its relative width, i.e., the width compared to the mean value, sharpens. Certainly, it is apparent that

$$\lim_{N\to \infty }N^{-1}S\left(\hat{\nu }\right)=0,$$

which shows that the relative randomness, i.e., the randomness per particle, becomes vanishingly small as N→∞. Meanwhile, the absolute randomness, represented by $S(\hat{\nu })$, increases as *N* becomes larger and larger.

In the end, the lack of formal specification of the physical role of microstates, together with the lack of consideration of the absolute randomness of macrostates, lead to the questionable belief that all the properties of a system of a large numbers of particles, including ”entropy,” depend on the macrostate $E\{\bar{n}\}$, because it contains ”the overwhelming majority” of microstates. From this standpoint, the subtraction of log(N!) is seen as a technical maneuver that becomes necessary to circumvent the challenge posed by the indistinguishability of particles. Note that the idea itself that one macrostate can contain the overwhelming majority of microstates is inherently questionable. This is because the ratio $|C^{N}\left(\bar{n}\right)\left|/\right|C^{N}|$ tends to zero as N→∞, regardless of the value of $\bar{n}$.

In conclusion, this paper made clear that, since the quantum state of a system of indistinguishable particles (bosons) is completely specified by the occupancy numbers of the quantum states allowed to system’s particles, the entropy the physical system is the Shannon entropy of the random occupancy numbers $\bar{n}$, which is obtained by subtracting the expectation of $\log(W\left(\bar{n}\right))$ from the entropy of microstates. Recognizing that $E\{\log\left(W\left(\bar{n}\right)\right)\}$ is the conditional Shannon entropy of microstates given the macrostate, we equivalently express the above concept by saying that the entropy of the physical system is equal to the mutual information between microstates and macrostates.

## Data Availability

No new data were created or analyzed in this study. Data sharing is not applicable to this article.
